# Abortion in Nepal: perspectives of a cross-section of sexual and reproductive health and rights professionals

**DOI:** 10.1186/s12905-019-0734-1

**Published:** 2019-02-26

**Authors:** Claire Rogers, Sabitri Sapkota, Anita Tako, Jaya A. R. Dantas

**Affiliations:** 1 International Health Programme, School of Nursing, Midwifery and Paramedicine, Curtin University, Perth, 6102 Western Australia; 2 Marie Stopes International, London, UK; 3 Independent Sexual and Reproductive Health Consultant, Bhaktapur, Nepal

**Keywords:** Abortion, Contraception, Family planning, Sexual and reproductive health and rights (SRHR), Nepal

## Abstract

**Background:**

Globally, women face many barriers in the attainment of sexual and reproductive health and rights (SRHR). Since 2002, the legalisation of abortion in Nepal has seen significant progress in the expansion of safe abortion and family planning services.

**Methods:**

This qualitative, exploratory study was conducted in 2014 and uses nine in-depth, open-ended interviews with a cross-section of SRHR professionals, to explore their perspectives on abortion in Nepal. The study was underpinned by the Assets Focused Rapid Participatory Appraisal (AFRPA) research methodology and used the health information pyramid conceptual framework.

**Results:**

Thematic content analysis revealed emerging themes relating to barriers to access and uptake of skilled safe abortion services and post-abortion family planning. Findings also emphasised current practical and legal components relating to the provision of medical abortion through pharmacies and highlighted issues of sex-selective abortion within the predominantly patriarchal society.

**Conclusion:**

Effective and ongoing sector-wide monitoring and evaluation of safe abortion services and their staff is essential for women in Nepal to have adequate access to effective and efficient safe abortion services, access to contraception and sexual and reproductive health (SRH) information post-abortion and to ensure adherence to current Safe Abortion Policy. It is critical that the unsafe (less and least safe) provision of medical abortion through pharmacies and sex-selective abortion continues to be investigated and that innovative strategies are formulated to ensure the cultural, reproductive and sexual health and rights of Nepali women are realised.

## Background

An estimated 56 million abortions occur each year worldwide, with women in developing countries having a higher likelihood of accessing abortion than their counterparts in developed regions [1]. World Health Organization (WHO) classifications of safe and unsafe abortion categorise abortion into a three-tiered model of safe and unsafe, with unsafe abortion being further divided into less safe and least safe [2]. It is estimated that of the total abortions occurring worldwide each year between 2010 and 2014, approximately 25.1 million were categorised as unsafe (17.1 million less safe and 8 million least safe) with 24.3 million, or 97%, of unsafe abortions occurring in developing countries [2]. Complications from unsafe abortions are especially common in developing countries with an estimated 6.9 million women annually requiring medical treatment after an unsafe abortion [1].

Prior to 2002, abortion was illegal in Nepal, unsafe abortion was common and deaths from abortion-related complications attributed to more than half of maternal deaths occurring in major hospitals [3–6]. Over the last two decades, Nepal has undergone a sector-wide government approach to family planning and safe motherhood, complementing the legalisation of abortion in 2002 and the availability of medical abortion (mifepristone and misoprostol) since 2009 [6, 7]. Through a concerted effort to reduce maternal deaths, Nepal has seen a dramatic decrease in its maternal mortality rate (MMR) over the last twenty years, declining from 901 per 100,000 live births in 1990 to 258 per 100,00 live births in 2015 [8, 9].

Data from the Nepal Department of Health Annual Report (2014/2015) demonstrates that since the implementation of safe abortion services, a total of 819,690 women have received safe abortions from certified service sites. However, the Department of Health Annual Report also details that utilisation of these safe abortion services has plateaued over the last few years [8]. The contraceptive prevalence rate (CPR) has also plateaued over recent years, with data from the most recent National Demographic Health Survey (NDHS), reporting a decrease in the use of modern contraceptive methods from 44% in 2006 to 43% in 2011 and remaining stagnate at 43% in 2016 [8, 10]. The unmet need for contraception in Nepal continues to remain high (having declined country wide from 32% in 1996 to 24% in 2016), particularly for married women aged 15–19 years (34.9%) and for women living in rural and remote regions of Nepal (25.3%) [10]. The low status of women, lack of education, poverty, isolation and the socioeconomic and sociocultural consequences of the caste system all remain significant barriers to SRH care access and service utilisation [10, 11].

In August 2016, the Government of Nepal announced a plan to implement free safe abortion services in public clinics, in combination with the provision of free family planning services, to help overcome the economic burden of accessing safe abortion services [12–14]. However, even with access to free services in public facilities, costs associated with transportation, accommodation, logistics, medicines and additional medical fees remain prohibitive factors for poor women accessing services, particularly those in rural and remote regions [4, 7]. Additionally, women seeking services through the non-government and private sectors are required to pay for safe abortion services out of pocket [7].

A Nepal based study of 527 women presenting at hospitals due to complications from unsafe abortion detailed that 68% of respondents induced termination through medication (any substance or drug taken orally or inserted vaginally) while 32% used instrumentation (insertion of instruments into the vagina including aspiration, dilation and curettage or foreign objects) [15]. An estimated 323, 000 abortions were performed in Nepal in 2014, and of these, over half (58%) are considered clandestine procedures, provided by untrained or unregistered providers or self-induced [16]. Of the estimated 137, 000 legal abortions performed in Nepal, the public sector provided 37% of procedures, 34% were provided at NGO facilities and 29% were obtained at private-sector clinics [16]. According to the most recent country-wide NDHS (2016), of the women who recorded having an abortion in the five years preceding the survey (*n* = 492), 72% opted for medical abortion (MA), 17%, manual vacuum aspiration and 7% dilation and evacuation/dilation and curettage. The majority reported attending a doctor, nurse, or auxiliary nurse midwife (71%) for their most recent abortion and 19% received services from a pharmacist or medical shop. Rural residents and women with no education were more likely to report the use of MA than their urban counterparts. Half of the women surveyed reported obtaining an abortion as they did not want more children, while 12% said that they wanted to delay childbearing. Health concerns were cited by 10% of women, 9% wanted to space their births, and 7% reported that the sex of the child was undesired [10].

While it has been 15 years since the legalisation of abortion in Nepal, unsafe abortion remains the third highest (7%) direct cause of maternal death in Nepal and significant numbers of Nepali women remain unaware of the legal status of abortion and have limited or no knowledge of where to obtain safe abortion services [7, 10, 17–19]. Efficient and equitable provision of Comprehensive Abortion Care (CAC), which includes Post-Abortion Care (PAC), plays a pivotal role in positive health outcomes and prevention of future unintended pregnancies for women who access safe abortion services [7, 20–25].

By providing a platform for SRHR professionals to share their extensive experience and knowledge, this qualitative exploratory study aims to enhance the current understanding of abortion in Nepal from their perspective and, to the authors’ knowledge, is the first of its kind to be conducted in Nepal with such a diverse group of professionals. Informed by participant suggested strategies, we propose recommendations on how SRH and CAC services can assist Nepali women to navigate cultural and sexual norms to more effectively and holistically exercise their reproductive health rights.

## Methods

Using an Assets Focused Rapid Participatory Appraisal (AFRPA) research methodology, underpinned by a health information pyramid conceptual framework, this qualitative exploratory study utilised data collected from in-depth, open-ended interviews with nine SRH professionals located in Kathmandu valley and Sunsari districts of Nepal [26, 27]. Building on the Rapid Participatory Appraisal methodology, Pepall et al. (2006) describe AFRPA as an effective means of prioritising needs and identify existing community assets that may help contribute to social change and development. Emphasis on assets ensures that findings are concurrently solution- and problem-focused, as opposed to merely the identification of problems. Supporting this methodology with the Annett and Rifkin’s (1995) health information pyramid conceptual framework, grounded data collection within a four-tiered framework: 1. community composition, organisation and structure, and capacity to act; 2. physical environment, socioeconomic environment, and disease and disability; 3. health and environmental services, and social services; and 4. health policy. Pepall et al. (2006) refer to this composite process as an Assets Focused Rapid Participatory Assessment Cycle (AFRPAC).

Throughout the research project, nine individual informal conversations were also conducted with community members located in the Sunsari District including: business owners; homemakers; political and religious leaders; academic professionals; and young people, to inform in-depth interview questioning and to enable the research team to acquire a greater understanding of people’s perception of women’s health within the context of their community, as well as Nepal in general. These informal conversations also helped to highlight perceived community and national assets, such as individuals, organisations, programs and infrastructure. As well as supporting in-depth interviews, this component of AFRPA allows researchers to gain insight into a community’s own perspective of its needs; helps facilitate the translation of overall findings into action; and assists in enabling the establishment and ongoing relationship between service providers and local communities [27].

Due to the cyclical nature of the AFRPAC, evaluation and modification of the research process were on-going. The utilisation of the AFRPA research methodology and health information pyramid conceptual framework enabled the evolution of information collection and analysis to accentuate key issues concerning the SRHR sector, as well as perceived assets relating to safe abortion and post-abortion family planning and SRH information access in Nepal. This enabled participants to focus both on current problems relating to access to safe abortion, post-abortion family planning and SRH information, as well as the formulation of potential solutions for these issues. Complementing the qualitative findings, analysis of current government and non-government SRH policy and clinical practices was also undertaken concurrently.

Ethical approval for this study was granted by the Nepal Health Research Council (NHRC 20/2014) and the Curtin University Human Research Ethics Committee (HR 17/2014). With assistance from in-country contacts within the SRHR sector, research participants were purposively recruited, via telephone and email, from a cross-section of professionals for their expertise within the SRHR field in Nepal. Representing varying SRHR organisations and professions, the diverse range of research participants included: clinical staff; researchers; advisors; advocacy officers; government officials; international and local non-government organisation staff; and international donor personnel. By design, many of the research participants are high-ranking officials or senior members within their organisations and were able to provide country wide context (urban, rural and remote) to SRHR and wide-ranging perspectives to SRHR in Nepal. While their organisations are represented throughout Nepal, many of the participants work from their organisational headquarters in Kathmandu, therefore the majority of interviews were held in Kathmandu from a practical perspective.

The hour-long interviews were conducted with participants (at a location of the participant’s choosing) between June and August of 2014 by the first and third authors who are both females. A research information sheet and informed consent form were provided to all participants for review prior to interviews and participants were encouraged to ask questions about the study or any component of the research. Participants choose either Nepali or English as their preferred language for communication. Informed written consent was provided by all research participants. Eight of the interviews were audio recorded, and one interview was transcribed on location. Audio files were transcribed after the interviews, and all participants received a copy of their interview transcript for review and feedback and as a form of member checking. A second round of member checks was conducted between June and July of 2017.

A thematic analysis of in-depth interviews was undertaken with the first three authors reading the transcripts and discussing data saturation before collaboration and refinement of themes. Thematic analysis enabled the examination of patterns of experience within the data, facilitating the synthesis of themes and sub-themes within the context of over-arching commonalities of access to safe, less safe and least safe abortion in Nepal [28, 29]. To ensure the confidentiality of participants, generic job titles have been used throughout this paper and the sex of the participants withheld.

Rigour in qualitative research is assessed within the context of dependability, credibility, confirmability and transferability, with trustworthiness a significant factor in the research’s ability to demonstrate reliability and validity [30–32]. To enhance the credibility and overall trustworthiness of this research, systematic checking, ongoing interpretation of data and an audit trail were utilised to ensure information relating to the study design, methods and analysis were documented, transparent and could be replicated [33–36]. Pilot testing of interview questions was conducted with all authors assisting in the refinement and finalisation of the guiding questions prior to the interviews. Member checking, the process of sharing interview findings with research participants to ensure data accuracy, was undertaken to validate results and themes and enhance the trustworthiness of the data [34, 36]. The second round of member checks conducted in 2017 was also an effective method of ensuing the continued relevance of respondent quotes [36]. Participants were also asked if they would like to provide additional information or insights into any developments in SRHR policy and practice in Nepal. As a component of the ongoing AFRPAC research process, the second round of member checks ensured findings are contemporary and relevant to issues impacting SRHR in Nepal.

## Results

The research participants worked within various sectors of SRHR in Nepal and provided extensive and multifaceted insights into their experiences and knowledge regarding women’s abortion and post-abortion experiences in Nepal. Eight participants were interviewed in Kathmandu, and to gain understanding of SRHR issues within a clinical environment outside of Kathmandu, one was interviewed in the Sunsari District (see Table 1).

**Table 1:** In-depth Interview (IDI) Participants (*n* = 9)

	Title	Organisation	Work Location
IDI 1.	Service Provider	SRH Clinic, I/NGO^1^(a)	Kathmandu
IDI 2.	Senior Service Provider	SRH Clinic, I/NGO(a)	Kathmandu
IDI 3.	SRHR Research Consultant	Independent	Nepal
IDI 4.	Senior Service Provider	CAC^2^ Unit, Public Health Facility	Kathmandu
IDI 5.	Safe Abortion Policy Advisor	SRH I/NGO(b)	Nepal
IDI 6.	Service Provider	SRH Clinic, I/NGO(a)	Sunsari District
IDI 7.	Program Manager	SRH NGO^4^(c)	Nepal
IDI 8.	Senior Officer	SRH NGO(d)	Nepal
IDI 9.	Senior Officer	Ministry of Health, GON	Nepal

^1^International Non-Government Organisation

^2^Comprehensive Abortion Care

^3^Government of Nepal

^4^Non-Government Organisation

(a) SRH I/NGO providing clinical and educational services

(b) SRH I/NGO providing advisory and educational services

(c) SRH NGO providing clinical and educational services

(d) SRH NGO providing research, advocacy and educational services

Three overarching themes emerged from the thematic analysis relating to 1. barriers to access and uptake of skilled safe abortion services, 2. barriers to access and uptake post-abortion family planning services (contraception and SRH information provision) and 3. the provision of MA through pharmacies. There were several sub-themes relating to each of the overarching themes and are listed in Table 2. Participants also recommended strategies to improve access and uptake of post-abortion family planning services and skilled safe abortion services in Nepal.

**Table 2:** Thematic Analysis of Interview Data

Main Themes	Sub-themes
Barriers: Access and uptake of skilled safe abortion services	
	Sociocultural attitudes
	Socioeconomic constraints
	Geographic isolation
	Translating policy into practice
Barriers: Access and uptake of post-abortion family planning services	
	Misconceptions and cultural barriers
	Geographic remoteness
	Policy, practice and monitoring
Concerns about medical abortion provision through pharmacies	
	The evolution of unsafe (less safe and least safe) abortion and medical abortion
	Obstacles to safe abortion services
	Challenges of monitoring unsafe (less safe and least safe) pharmacy provided medical abortions

### Barriers: Access and uptake of skilled safe abortion services

#### Sociocultural attitudes

Participants highlighted the impact of sociocultural attitudes, religious beliefs, cultural norms relating to sexual and reproductive health and the influence of Nepal’s patriarchal society on women’s access to and uptake of skilled safe abortion services. Stigma associated with accessing safe abortion services was stated as a key barrier to women not utilising safe abortion services.

Stigma in the community is the first barrier to women accessing safe abortion services. A woman can say that she is going to a health service for a delivery, but she can’t say she is going for abortion services. *IDI 5. Safe Abortion Policy Advisor, I/NGO, Nepal.*

Negative service provider attitudes towards women seeking safe abortion services were also expressed and this impeded woman from up taking services. Several participants stated that unmarried women have further increased difficulties in accessing and use of safe abortion services due to negative cultural attitudes towards sex outside of marriage. One participant stated that this impacts women who often seek unsafe abortions.

Although abortion is legal in Nepal, many women are still getting unsafe abortions; we see less unmarried women attending safe abortion services (due to sociocultural reasons). *IDI 6. Service Provider, SRH Clinic, I/NGO, Sunsari District.*

#### Socioeconomic constraints

The complex socioeconomic disparities between women who are married and unmarried women seeking safe abortion services compound barriers to access.

Barriers exist between married and unmarried women seeking abortions…The majority of women using safe abortion services are typically from economically relatively well-off families. Poor women experience a greater financial barrier to accessing abortion services. *IDI 3. SRH Research Consultant, Independent, Nepal.*

The financial burden experienced by women seeking safe abortion services (including appointment and procedure fees as well as travel) was also a reoccurring theme throughout the interviews. Several participants stated that the cost of safe abortion services deters women from accessing these facilities and is a deciding factor for many women to procure unsafe abortions. The inability to afford safe abortion services is often exacerbated for women living in rural and remote regions of Nepal.

Another barrier to access and uptake of skilled safe abortion services is the abortion fee… 1000 Nepali Rupees ($9.50 USD) is a very big amount for rural women. *IDI 8. Senior Officer, SRH NGO, Nepal.*

#### Geographic isolation

The health services in Nepal are located in the Central Development Region and along the Terai belt (the low land region in Southern Nepal), to the detriment of those living in the remote hill and mountain regions.

In Nepal, approximately one in five people live in urban areas where both surgical and MA services are available. In rural areas and districts, however, access to abortion services remains problematic. In such areas, women may not have any option but to resort unsafe abortion practices that could have serious health consequences. *IDI 3. SRH Research Consultant, Independent, Nepal.*

Issues relating to lack of access to health care services, safe abortion services and trained medical professionals in rural and remote regions was a reoccurring theme throughout the interviews.

I come from the rural area, but I don’t choose to work in my hometown because I cannot earn as much money providing services there… Few health professionals remain in remote areas and very few I/NGOs are providing training for those professionals. *IDI 2. Senior Service Provider, SRH Clinic, I/NGO, Kathmandu.*

Interview participants revealed numerous inhibiting factors relating to lack of access and uptake of safe abortion services in rural and remote regions of Nepal including: lack of infrastructure, both health services as well as road and transportation issues; administrative issues relating to lack of sufficient numbers of trained staff able to provide non-judgmental safe abortion services during regular working hours; lack of training and on-going capacity building of services providers; and lack of incentives for trained personnel to work within rural and remote regions.

#### Translating policy into practice

Participants reported their experiences with the conflicting nature of practice versus the Ministry of Health National Safe Abortion Policy relating to effective and equitable SRH service provision in Nepal.

I think the written policy is very good, but in practice, it’s not applicable…The Government has a concrete plan of action, but they lack the knowledge and skills on where to implement and how to implement. *IDI 7. Program Manager, SRH NGO, Nepal.*

While current Government policy states that safe abortion services are available across Nepal, several participants stated that lack of awareness of abortion laws and available services continues to inhibit women from access and uptake of safe abortion services.

CAC services are available in all ecological areas; however, due to difficult geographical locations, women are facing difficulty in accessing services. Also, many women in these remote locations are not aware of safe abortion services. *IDI 4. Senior Service Provider, CAC Unit, Public Health Facility, Kathmandu.*

Geographic isolation was stated as being a deterrent for trained health care professionals, who do not want to work in remote and rural regions.

The Government has health policy that clearly states the need for doctors and medical staff in remote areas, but the incentives are very low. In public facilities, a trained medical doctor and nurse positions are allocated up to the Primary Health Care Centre (PHCC) level, but the positions are often vacant. *IDI 7. Program Manager, SRH NGO, Nepal.*

Participants reported that monitoring and evaluation mechanisms of safe abortion services to maintain quality of care and ensuring CAC service data is accurately recorded and reported, are inconsistent across public, I/NGO and private services.

The main cause of inconsistency is the lack of a monitoring mechanism… There are no regular monitoring visits in some NGOs as well as in the public sector. Monitoring is very weak. *IDI 5. Safe Abortion Policy Advisor, I/NGO, Nepal.*

Effective and consistent monitoring and evaluation processes of I/NGO safe abortion services in Nepal was reported to be currently undertaken within I/NGO SRH clinics. However, lack of trained professionals to conduct monitoring and evaluation of safe abortion services provided by public and private facilities was sighted as being the key component of inconsistent services provision, impacting women’s ability to access and utilize safe, effective and comprehensive abortion services.

Here (Government Office) we are only two staff members, one doctor and one Public Health Nurse (PHN), we can’t go to every district to monitor sites so (SRHR I/NGO) is supporting the Government with monitoring. In the districts, we have a PHN as a focal person who should monitor the district hospital and other private clinics. She should go from time to time (to monitor services), but in some districts, it is lacking. It depends on how active PHN is. *IDI 9. Senior Officer, Ministry of Health, GON, Nepal.*

Current translation of policy into practice and monitoring of safe abortion services in Nepal are intrinsically linked and have a substantial impact on access to safe abortion services with trained and culturally sensitive health professionals. Increasingly, the Government is establishing a memorandum of understanding (MoU) with I/NGOs to overcome the lack of resources and expertise they lack for monitoring and evaluation of safe abortion services. While the vast majority of participants shared their positive perceptions of Government SRH and Safe Abortion Policy in Nepal, lack of effective monitoring inhibits the translation of policy into practice.

It’s not so much the policy, rather the implementation that must be looked at. Implementation of the guidelines needs to be monitored… The Government needs to have a regulating body to assess, evaluate and monitor the standard of abortion provision services. *IDI 3. SRH Research Consultant, Independent, Nepal.*

### Barriers: Access and uptake of post-abortion family planning services

#### Misconceptions and cultural barriers

In Nepal, contraception is free of charge and consequently, socioeconomic issues relating to access and uptake of post-abortion contraception did not emerge in our interviews. However, sociocultural factors impacting post-abortion contraception decision making were a prominent theme discussed by all participants. Prevailing misconceptions relating to the use of modern contraception was a critical factor in women deciding not to use post-abortion contraception.

There are many misconceptions regarding the use of contraception like excessive bleeding will occur, weight gain will happen, or they will get cancer through the use of contraceptive methods. *IDI 6. Service Provider, SRH Clinic, I/NGO, Sunsari District.*

Effective provision of post-abortion SRH information and contraceptive education through counselling was stated as an important component of increasing uptake of post-abortion contraception. However, challenges were reported.

Good counsellors have key role to play in reducing the misconceptions around contraception. If a counsellor is well trained and knowledgeable, they will motivate the client to use contraception and provide comprehensive information. Half information leads to misconception. *IDI 7. Program Manager, SRH NGO, Nepal.*

The difficulty of discussing post-abortion contraception support and services with husbands and mothers-in-law is a cultural barrier.

Cultural and social barriers are the most common barriers for women accessing post-abortion contraception….in some cases, women still cannot share their SRH related problems with their husbands. *IDI 1. Service Provider, SRH Clinic, I/NGO, Kathmandu.*

While our participants shared many examples of barriers to uptake of post-abortion contraception and SRH information, the most frequently cited inhibiting factor to the uptake of post-abortion family planning services was the large number of women whose husbands work outside of Nepal or away from their home districts.

In our country, mostly the males (husbands) are migrant workers, who occasionally come home to visit their wives. That is one of the main reasons for women not accepting contraception (post-abortion). *IDI 5. Safe Abortion Policy Advisor, I/NGO, Nepal.*

All participants shared their thoughts relating to the ever-increasing male migrant worker population and the impact it is having on women using contraception and post-abortion contraception.

#### Geographic remoteness

Issues with timely procurement and supply of contraceptives was also a barrier to women receiving a broad choice of contraceptive options post-abortion. Reported contraceptive supply chain challenges include lack of trained logistical staff and insufficient human resources; lack of adequate storage and timely transportation from district centres to peripheral health facilities; and an absence of accountability mechanisms for stockouts and commodity delays. Along with a lack of supply, geographical isolation also impacted the availability of trained safe abortion service providers able to provide women with comprehensive post-abortion family planning services and SRH information.

At rural and remote sites (safe abortion services), contraceptive commodity and trained human resources are not always available. If a woman comes to the service site and wants an Implant, due to lack of trained human resources and commodity she can’t get an Implant. That gap of commodity and trained staff means women are not getting contraceptive method of their choice and the counselling will be biased in that case. The service provider will counsel on those methods which are available at the site. *IDI 5. Safe Abortion Policy Advisor, I/NGO, Nepal.*

#### Policy, practice and monitoring

Participants spoke of the juxtaposition between Government policy that requires clinics to have five different methods of contraception available to clients and the reality of the limited choice, perhaps only two or three contraceptive methods, available at many public clinics.

According to Government Policy, at the Health Post level, there must always be the availability of five contraceptive methods (condom, pills, Depo (injectable), IUD and implant). But, there are not all five methods available at all the government Health Posts in Nepal. *IDI 9. Senior Officer, Ministry of Health, GON, Nepal.*

While the current Government policy states all SRH facilities should follow the same guidelines, service provision continues to vary due to poor contraceptive supply, lack of trained service providers and a high level of staff turnover resulting in clinic closures. This lack of comprehensive education and knowledge provision among health professionals across facilities was reported to be a key inhibitor of post-abortion contraception uptake and continuation.

Women need to be informed and educated so they can make an informed decision…Most abortion clinics offer contraception in an almost a ritual way… Through counselling and discussion, the service provider must ensure that the women’s individual contraceptive needs are met… Counsellors should really focus on the women’s fertility goals and desires and see what the best way for her to achieve that is. *IDI 3. SRH Research Consultant, Independent, Nepal.*

Although current Government SRH and Safe Abortion Policy addresses the SRHR needs of Nepali women on paper, it was emphasized there is ineffective translation and application of policy into practice.

In Nepal, implementation of policy into action leaves a lot to be desired. *IDI 3. SRH Research Consultant, Independent, Nepal.*

#### Concerns about medical abortion provision through pharmacies

#### The evolution of unsafe (less and least safe) abortion and medical abortion

All interviewees commented on the increasing trend of women accessing both registered (Government approved) and unregistered brands of mifepristone and misoprostol (MA pills) illegally through pharmacies, sometimes referred to as chemists or medical shops. Respondents also raised concerns that not only are registered and unregistered MA tablets sold through pharmacies, but potentially drugs of unknown chemical composition are also being provided to induce abortion. One participant elucidated the evolution of unsafe abortion in Nepal and its impact on negative health outcomes for women.

Before the legalization of abortion, women practised harmful abortion methods such as taking herbs and extensive massage which could cause complication and the need for hospital admission. Now, because of the availability of abortion pills, women go to pharmacies, take the pills, have incomplete abortion or complications and need to go to the hospital. *IDI 8. Senior Officer, SRH NGO, Nepal.*

Several participants reported that a high number of women accessed safe abortion services after experiencing an incomplete abortion as a result of accessing MA illegally through pharmacies.

Most of our incomplete abortion cases come from medical shops. *IDI 4. Senior Service Provider, CAC Unit, Public Health Facility, Kathmandu.*

The participants shared the reality of women accessing MA through pharmacies and the lack of SRH information relating to the administration of MA as well as the lack of post-abortion care and post-abortion family planning.

Pharmacy staff are not aware of the eligibility criteria for the provision of abortion services, what is the route of administration, what is the expected outcome and side effects, what are adverse side effects. They are just selling medical abortion pills. *IDI 8. Senior Officer, SRH NGO, Nepal.*

Several reasons for women choosing to access abortion through pharmacies instead of going to health facilities for safe abortion services were cited during the interviews. Negative sociocultural attitudes towards abortion and privacy issues relating to accessing abortion were reported to be a key reason why women chose pharmacies.

Abortion is very stigmatized in Nepali culture, so they scared of losing their privacy… in chemist shop they don’t have to give any answers regarding the abortion they can get pills very easily. *IDI 5. Safe Abortion Policy Advisor, I/NGO, Nepal.*

#### Obstacles to safe abortion services

Geographical isolation and the health service provision implications were also reported as key influences in women choosing to access MA through pharmacies. Participants shared that women will often seek MA through pharmacies as there is no health facility that provides safe abortion service located near them or there are no trained safe abortion service providers in their local community.

The reason for women choosing medical shops over safe abortion services (from registered clinics) may be due to the lack of abortion services in government health facilities in their area. *IDI 8. Senior Officer, SRH NGO, Nepal.*

#### Challenges of monitoring unsafe (less and least safe) medical abortion

Participants spoke of numerous challenges when trying to stop the sale of MAs through pharmacies in Nepal.

In current Government policy, it is stated that medical abortion should not be provided through chemist shops, it should be provided through permitted (registered) clinics only. But the demand is very high…The clients don’t care about World Health Organization (WHO) standards and protocol, they just want prompt service. *IDI 5. Safe Abortion Policy Advisor, I/NGO, Nepal.*

The difficulties the Government Drug Administration Department faces trying to monitor and halt the flow of MA pills through pharmacies was reiterated throughout the interviews. Human resource capacity was cited as being a major barrier to the effective monitoring of MA drugs within the market.

Medical abortion obtained from medical shop should stop. We have approached the Department of Drug Administration but they can’t control it, and this is a great concern. *IDI 9. Senior Officer, Ministry of Health, GON, Nepal.*

Several spoke of collaboration between Government, I/NGOs and the private sector to establish a committee to implement monitoring of pharmacies. However, this has proven challenging with inconsistent results seen across areas of implementation. While MA can only legally be prescribed by a safe abortion service provider in the first trimester in Nepal, it was suggested that women might have a sex-selective MA through pharmacies after the first trimester.

There are currently no regulatory body on sex-selection issues in Nepal. Technology (ultrasound) has made it easier for couples to test for the sex of their child… It needs to be audited and evaluated; sex-selection is emerging as a big concern with the increase of accessibility to medical abortion as well. The MoH (Ministry of Health) needs to set up an independent body to ensure that ultrasound is not used for sex-selective abortion in the country. *IDI 3. SRH Research Consultant, Independent, Nepal.*

One participant stated that within the context of sociocultural practices and beliefs, the motivation behind sex-selection abortion and the legalization of safe abortion (both surgical and medical), should be not be viewed as mutually dependent. They commented that the legalization of abortion in Nepal is not the causation factor of sex-selection abortion practices but is a component of entrenched cultural beliefs.

Sex selection is related to the social, economic and patriarchal pattern of society. It has been done for many years prior to the legalisation of abortion. Linking the legalisation of abortion services and sex selection is wrong. If we educate the community people on the value of the girl child, if boys and girl are equally valued, sex selection abortion will decrease, it takes time, it won’t change overnight. *IDI 5. Safe Abortion Policy Advisor, I/NGO, Nepal.*

Interviewees shared their views on the current state of MA provision in Nepal and on how unsafe provision of MA can decrease.

Medical abortion has left the clinic, left the doctors. Technology has outpaced everything, so we have to find a way to engage the non-conventional facilities. Medical shops are increasing medical abortion provision; however, they are just dispensing the drugs and are not counselling the women… Medical shops are evolving their role in Nepal. Globally, the function of medical shops has enlarged and is recognized by the World Health Organization (WHO) as well. *IDI 3. SRH Research Consultant, Independent, Nepal.*

While one research participant stated that MA pills should only be administered by professionals that are able to perform pelvic or vaginal examinations, several participants shared their views of a more harm reduction approach through education and training of safe MA provision to pharmacy staff.

For medical shops that don’t have medical professionals, we should provide at least basic harm reduction training. *IDI 7. Program Manager, SRH NGO, Nepal.*

Several participants highlighted the need for Government policymakers to carefully consider the role pharmacies currently play in the provision of MA and how current policy must be revised to reflect this.

(The Government of Nepal) should revise policy as we can’t stop chemist shop from selling the pills (medical abortion). If more restrictions are made, they will, of course, sell the pills under the table… Rather than stopping chemist shops and stopping women visiting chemist shops we should give information on the right dose and right time or complete information of taking pills in case of abortion. *IDI 5. Safe Abortion Policy Advisor, I/NGO, Nepal.*

Pharmacy specific harm reduction strategies such as training of non-medical staff, as well as the potential for qualified pharmacy staff receiving further education on MA provision through pharmacies, was noted as a potential governmental approach to increase access to safe MA as well as theoretically reducing the incidence of complications resulting from unsafe MA provision.

Through training, medical abortion services can be expanded, and those who are eligible to provide medical abortion could provide services. For those who don’t meet the edibility, their business selling medical abortion will decrease, and therefore complications of medical abortion will be reduced. *IDI 8. Senior Officer, SRH NGO, Nepal.*

The certainty that pharmacy staff will continue to sell MA tablets regardless of illegality was reiterated by research participants. The majority of participants also stated the need to review and revise current government policy relating to MA provision through pharmacies to help reduce mortality and morbidity associated with the practice.

## Discussion

The intersectoral and multifaceted nature of barriers associated with access and uptake of skilled safe abortion services and post-abortion family planning are highlighted throughout our findings. Insight and knowledge shared by the SRHR professionals is underscored with recommendations on ways to improve access and uptake of skilled safe abortion services and post-abortion family planning, as well as strategies to decrease the level of mortality and morbidity. Our findings emphasise the breaking down of sociocultural stigma and entrenched cultural beliefs and gender inequality, related to abortion and contraceptive use, as key to supporting women to access and uptake safe abortion services and post-abortion family planning, a concept collaborated with previous SRHR research [22, 37–39]. While the Government of Nepal has focused on improving access to health care services through the strengthening community-based health interventions [40], our findings demonstrate the continued gap in community-level safe abortion and family planning education and knowledge.

Through collaboration with I/NGO and private sector, the Government of Nepal must increase awareness campaigns relating to access and uptake of safe abortion and family planning services at the community and national level. Within the *Nepal Health Sector Strategy 2015–2020* framework, it is essential that community-based SRHR and safe abortion education and awareness campaigns focus on families, women, males, youth, community leaders and service providers [17, 40–42]. The need for policy to address the status of women within Nepal’s patriarchal society is recommended as a means to ensure women’s SRHR, as well as their human rights, are met.

Several respondents reported the increasing use of sex-selective abortions and the sociocultural attitudes that prefer a male child that are responsible for this practice. Indeed, there has been global research into the consequence of son-preference and sex-selective abortion, most notably in China, South Korea and parts of India [43]. Currently in Nepal research on sex-selective abortion remains scare and is needed to inform community educational and behavioural change interventions and policies [44]. Increased activities addressing and shifting culturally entrenched attitudes towards girls and women within Nepali society must increase [45].

In the last decade, Nepal has experienced an unpredicted level of male workforce migration, both within Nepal and to overseas countries, predominantly The Middle East and India [10, 40, 46, 47]. Several studies conducted in Nepal have demonstrated that the increasing male migrant workforce could potentially play a role in Nepal’s stagnated contraceptive prevalence rate, highlighting a key factor to whether women decide to access, continue and/or discontinue contraception is greatly influenced by issues relating to spousal separation [11, 22, 37, 39, 48–50]. SRH policy and practice stakeholders must establish strategies to decrease the cultural stigma surrounding women accessing contraception while their husbands work away from the family home and to ensure service providers effectively counsel women on contraceptive choices that suit their circumstance. More research into the association between contraceptive use among women and the migrating male workforce is needed.

Supporting our findings, previous studies show that knowledge on the legalisation of abortion in Nepal and the awareness of where to access safe abortion services remains low, particularly for young women, women from rural or remote regions and for women from lower socioeconomic backgrounds [17, 41, 42, 51]. Misconceptions and myths related to family planning (such as fear of side-effects) have been shown to play an inhibiting role in a woman’s decision to uptake post-abortion contraception [52]. Effective and comprehensive abortion and post-abortion counselling skills enables providers to support women’s SRH information and education needs; dispel misconceptions and discuss concerns relating to contraceptive use and side-effects; encourages discussion of fertility goals; facilitates referral to other health services if needed; encourages post-abortion follow up; and assists women in making informed decision regarding post-abortion contraception [22, 23, 37, 39, 52, 53].

The importance of effective and equitable training and capacity building for all SRH professionals, regardless of geographic location or type of clinic they work in (public, private or I/NGO), is an important component in the provision of high-quality, safe abortion services and was a reoccurring recommendation throughout our research. The provision of adequate staffing numbers and positive service provider attitudes are important components of high-quality, safe abortion care and effective post-abortion counselling [22, 37]. The recruitment and retention of trained health care providers working in rural and remote facilities in Nepal continues to be a challenge across all sectors of health [40, 54, 55], with our findings highlighting the specific concerns relating to safe abortion services.

While the National Safe Abortion Policy states that safe, accessible and affordable abortion services should be ‘available with equity and equality for all women’, many Nepali women still do not have adequate access to such abortion services [4, 5]. This key finding corresponds with findings by Puri et al. (2016), that despite expansion of safe abortion services in Nepal, there remains a vital need to increase access and availability to high-quality, safe abortion services to all Nepali women, regardless of geographical location. The Government of Nepal, I/NGO and private practice SRH services must re-evaluate current incentives and strategies for health professionals to retain them in rural and remote regions [56]. Collaboration with universities and medical training facilities is recommended to formulate strategies to increase the number of trained health professionals choosing and continuing to work in rural and remote regions.

Women in Nepal face numerous socioeconomic barriers to accessing safe abortion services, particularly those who live in rural and remote regions. To help mitigate the financial barriers to accessing safe abortion services, the Government of Nepal has recently committed to provide free access to safe abortion services from public health facilities [12, 13]. However, without effective monitoring and evaluation of safe abortion services that are already being provided in public health facilities, ensuring a high level of quality care in these services remains impossible [57]. Therefore, a strategy for consistent and comprehensive monitoring and evaluation of these services must be rolled out simultaneously for this implementation to truly provide effective and equitable provision of comprehensive safe abortion services.

The WHO *Safe Abortion: Technical policy guidance for health systems* (2012) emphasises that quality safe abortion care depends upon effective operational processes for monitoring, evaluation and the effective implementation of rights-based SRHR policy. Along with maintaining quality of care, the accurate collection and reporting of service statistics are essential for the analysis and synthesis of population data relating to abortion in Nepal [5, 6, 57]. In Nepal and around the world, there is an urgent need to increase the availability of accurate information on gender equality and women’s and girls’ SRHR to inform rights-based policy and decision making. [45, 58, 59]. Effective monitoring and evaluation of safe abortion service facilities and health care providers is essential to facilitate a quality of care standard throughout Nepal and to ensure: adequate numbers of staff are located at clinics to deal with work flow; up to date training and maintenance of professional competency of all staff; administration and infrastructure is maintained to policy standards; and equitable distribution of contraceptive commodity supplies through an established and well-resourced supply chain.

Similar to findings of previous SRH research in Nepal, our participants expressed concern over the differing levels of consistency (between public, private, I/NGO in urban/remote areas) in providing access to a broad choice of contraceptives as well as effective family planning information regarding a broad range of methods [6, 22, 37, 50]. USAID’s 2016 report *Twenty-Five Year Review of Assistance to Nepal’s Health Sector* indicates the Nepal health sector procurement system and supply chain management remains weak throughout the health system, resulting in resource inefficiencies and frequent stock-outs of drugs and commodities at health facilities [47]. Our findings highlight the specific and current impact ineffective contraceptive commodity supply chains has on family planning services, particularly in rural and remote regions. Ensuring the close monitoring of commodity supply to facilitate the effective and equitable distribution of contraceptives, especially to remote regions, is a key element to ensuring uptake and access to post-abortion contraception services [60].

The provision of MA in the form of mifepristone and misoprostol where MA is legal (or misoprostol alone where mifepristone is not legal or available), is a proven, acceptable, safe and effective way to terminate an unwanted pregnancy up to nine weeks of gestational age [56, 57, 61]. Studies from countries where abortion is highly restrictive have credited, at least partially, a decline in rates of severe complications and mortality resulting from unsafe abortion with increased use of misoprostol [62–67]. While data is still limited, global research indicates the effectiveness of MA self-management and remote support (telephone hotlines or mobile phone messages) [67–71]. Current WHO guidelines do not recommend pharmacy workers and lay health workers independently provide MA due to insufficient evidence of safety and effectiveness [72]. Indeed, studies conducted in low and middle-income countries have demonstrated that without professional development or training of staff, women accessing MA through pharmacies rarely receive adequate information regarding the administration of the drug, post-abortion care, SRH information, post-abortion family planning or any form of follow-up [56, 73–77]. However, with appropriate training, several studies have documented that pharmacy workers and lay health workers are able to: assess clients for MA eligibility; provide adequate information on MA administration; provide information on, and management of side-effects; assess for abortion completion; provide post-abortion contraceptive and capably provide clinic-based referral when needed [66, 73, 78–81].

Our findings indicate there is a potential impact MA provision through pharmacies is having on mortality and morbidity from unsafe abortion in Nepal, and recommend harm reduction strategies, such as training pharmacy staff in safe MA provision and post-abortion family planning and care referral mechanisms, to decrease complications [73, 80, 82]. Respondents shared that the professional development training and certifying of eligible pharmacy staff in the provision of MA could not only decrease unsafe abortion and its potential consequences but also increase access to MA, particularly in remote and rural settings that currently lack safe abortion facilities [73, 83–85]. With increasing access to pharmacy provided MA in the developing world, our findings highlight the importance of harm reduction strategies to decrease mortality and morbidity from unsafe abortion, even within countries with permissive abortion laws [7, 67, 81].

It is essential that the Government of Nepal acknowledge the role pharmacies currently play in the provision of MA and establish practical strategies and policies to decrease negative health outcomes for women [80, 82]. A comprehensive evidence base relating to MA provision through pharmacies is needed to inform effective policy within Nepal. Further research is also necessary to differentiate the proliferation of MA distribution through pharmacies under current laws underpinned by the WHO framework of Safe Abortion, Less Safe Abortion and Least Safe Abortion [2].

Participants in this study recommended that through collaboration of public, I/NGO and private practice, SRHR policy and programmatic issues must be looked at critically to ensure robust and proactive policy for family planning and safe abortion service provision. While our findings suggest SRHR professionals are highly supportive of current National Safe Abortion Policy in Nepal, the need to translate and implement this policy into practice is still yet to be achieved [5, 6]. Within the country’s recently drafted Sustainable Development Goals strategies [45], the omission of specific indicators addressing safe abortion service access and uptake in Nepal seems a missed opportunity, particularly in the light of the country’s globally lauded success of legal safe abortion service implementation over the last 15 years [86–88].

While every effort was made to mitigate bias within the study and to enhance credibility and trustworthiness, the study has several limitations. Financial and time constraints for this exploratory study restricted time in the field. Practicalities relating to access of participants meant the study was limited to the Kathmandu area and did not include multiple participants from outside the region from government and non-government backgrounds. Due to time restraints and access, SRH clinic-based service providers were only sampled from one SRH I/NGO (one Senior Service Provider and two Service Providers) and one Government facility (one Senior Service Provider). However, data saturation was achieved with our small number of participants and enabled detailed analysis. As this is a qualitative study, we have ensured that the depth and richness of information collected, and an audit trail, will ensure transferability.

## Conclusion

Nepal’s change from restrictive abortion laws to liberalisation in 2002, and the country’s experience in expanding safe abortion services over the last 15 years, offers significant lessons for other low-and middle-income countries seeking to reduce mortality and morbidity from unsafe abortion [85]. However, as demonstrated within the Nepal context, even in countries where abortion is legalised, unsafe abortion is practiced. This cross-sectional, exploratory study highlights the numerous factors impacting the access to and uptake of safe abortion services and post-abortion family planning. These factors hold global applicability within in other resource-poor setting like Nepal. Sociocultural, socioeconomic and geographic barriers have highlighted the difficulties women in Nepal face when accessing safe abortion and post-abortion family planning services.

Our findings suggest that without effective and ongoing sector-wide monitoring and evaluation of SRH and safe abortion services and their staff, not all women in Nepal will have adequate access to quality safe abortion services and post-abortion family planning. It is vital that issues relating to the least safe provision of MA through pharmacies and sex-selective abortion continue to be investigated with innovated strategies formulated to ensure the sexual and reproductive health and rights of Nepali women are realised. Our findings detail the necessity for the translation of current Safe Abortion Policy into practice and for safe abortion service access and uptake to play a more prominent role in ongoing Nepal Health Sector Strategies and Sustainable Development Goal frameworks.

## Abbreviations

AFRPA: Assets Focused Rapid Participatory Appraisal
AFRPAC: Assets Focused Rapid Participatory Assessment Cycle
CAC: Comprehensive Abortion Care
MA: Medical Abortion
PAC: Post-Abortion Care
SRH: Sexual and Reproductive Health
SRHR: Sexual and Reproductive Health and Rights
